# Retraction: miR-133b inhibits glioma cell proliferation and invasion by targeting Sirt1

**DOI:** 10.18632/oncotarget.28678

**Published:** 2024-12-27

**Authors:** Chuntao Li, Zhixiong Liu, Kui Yang, Xin Chen, Yu Zeng, Jinfang Liu, Zhenyan Li, Yunsheng Liu

**Affiliations:** ^1^Department of Neurosurgery, Xiangya Hospital of Central South University, Changsha, 410008 Hunan, China

**This article has been retracted:** After concerns were raised on Pubpeer, the authors contacted Oncotarget, stating that ‘Our institution had several core facilities which we shared some equipment with many researchers during the study process. We cannot exclude the possibilities and leakages of data management leading the aforementioned concerns. Responsibly, we found that the statistical analysis of transwell was inaccurate.’ Additionally, the authors ‘repeated the experiments multiple times. The results presented consistently support the conclusion. We do believe that our study and conclusion are reliable.’ Oncotarget contacted the authors’ institution to request an investigation regarding the ‘data management’ issue, but received no reply. Oncotarget’s internal review found that based on the nature of the duplications (transwell assay and western blots do not need any facility, they are the simple assays), the authors’ request to make corrections with repeated experiments was invalid. The transwell assay data found in Figures 3B and 4C were used in four papers published previously that have all since been retracted: Figures 3B and 6B of [1], Figure 4C of [2], Figures 2C of [3], and Figure 3, panels C and D of [4]. In light of these facts, the Editorial decision was made to retract this paper.

Original article: Oncotarget. 2016; 7:36247–36254. 36247-36254. https://doi.org/10.18632/oncotarget.9198
